# Dr. Hammond’s Reply to Dr. Hopkins

**Published:** 1884-11

**Authors:** William A. Hammond

**Affiliations:** 43 West Fifty-fourth Ssreet, New York


### DR. HAMMOND'S REPLY TO DR. HOPKINS.

43 West Fifty-fourth Sreet, New York, Oct. 10, 1884.

*To the Editors of the Atlanta Medical and Surgical Journal*—

Gentlemen: On the 19th of August last, Dr. J. G. Hopkins brought to me a young gentleman, whom he introduced to me as his cousin, and who he informed me had come for medical treatment. Upon examination I found that he was suffering from certain mental and physical symptoms. The mental symptoms were depression of spirits and a "morbid fear" that if he was deprived even for a few minutes of personal association with Dr. Hopkins, something terrible would happen to him. If the Doctor left his sight he was at once seized with the most overpowering fears and horror, and a feeling that he could not accurately describe. This condition had lasted several months, and was of so intense a character that the young man had left his father's house and had gone to live with the Doctor. It is a well-known form of mental disease. His physical symptoms consisted of pain in the head, dizziness, a sensation of constriction around the cranium, and gastric and intestinal derangement.

These were the essential facts ascertained by a conversation with the young man and with the Doctor. As is my usual habit I examined a drop of his blood with the microscope. I was mainly in this course desirous of ascertaining whether or not there was uric acid present in it. He then, accompanied by Dr. Hopkins, went into an adjoining room where I examined him with the ophthalmoscope, "the large double convex lens and a circular peforated reflector," mentioned by Dr. Hopkins, an instrument that he does not appear ever to have seen before—and then with Lombard's thermo-electric differential calorimeter, an apparatus that has been over and over again described in medical journals and books, and which I have employed for over ten years past, and which is in use by other physicians of this and other cities. As the result of this part of the examination, I came to the conclusion that the patient was the subject of passive cerebral congestion, mainly involving the basilar surface. I cauterized the nape of the neck, applied statical electricity to the same part and the cervical and dorsal regions generally, advised the use of ice to the nucha, and gave internally a mixture of bromide of sodium, pepsin, powdered charcoal and water, and directed him to return the following day. He did not do so, but the four following days he came, accompanied by the Doctor, so that I saw him five times, for which he paid me my customary fees of twenty dollars for the first visit, and ten for each subsequent one, making sixty dollars in all. When he left the city he reported himself to me as being decidedly better, and he was better. He had been able to dispense with the Doctor's society for several hours at a time, and his head symptoms were alleviated. I did not promise to cure him, for I never made such a promise in my life. I did not treat him discourteously, for that is not my way, as many patients of mine in Georgia will freely state. I did on one occasion speak harshly to him because on the day before he had not sufficiently exerted himself to do without the society of the Doctor, but this was my duty.

I used no secret, unusual, or unprofessional methods of examination and treatment. They are all laid down at length in my work on diseases of the nervous system and have been explained every year to my medical classes in my lectures. If they were new to Dr. Hopkins, that I submit, is his fault, not mine.

As to my fees, I leave them on the table through the morning as they are paid by patients. I have no family practice and most of those who consult me prefer to pay for each visit as is a common practice in this city. I had several first patients every day, and there were, of course, several twenty dollar bills on the table every time Dr. Hopkins came to my house.

I charged this patient exactly what I have always charged during the last twenty years, and as every patient I have had from Georgia will testify for himself or herself, they are my customary charges, and I do not abate them except in the cases of patients who cannot pay them and with clergymen. They are no larger than those of many other physicians in this city.

In regard to the feigned operation that the Doctor says he heard of from some "New York doctors," I demand the names of these slanderers, when I promise to give them an opportunity of making their allegations good.

As to my being "fired out of the United States Army," that is true, but he very unfairly neglects to state that five years ago I was "fired" back to my old place with all my old rank, and that I am still in the army. I expect to stay there as Surgeon General and Brigadier General on the retired list as long as I live. The warning to "Southern physicians" will doubtless fall on heedless ears. I am a "Southern physician" myself and my Southern brethren know me too well to believe a slander.

As to sitting in an antique chair, having a frescoed consulting room, 3,000 volumes in my medical library, (5,000 he ought to have said), a clock that strikes hours and half hours and chimes hourly, a "gorgeous poly-colored chandelier" and a "variety of other things too numerous to mention," it will scarcely be necessary for me to say anything. If he could get them and had the taste to enjoy them he would probably indulge his fancy. His accurate knowledge of the amount and character of the bills on my table could not have been obtained unless he had fingered them during my absence from the room in making analysis of the urine of the patient, which I did every day except the first. To do this he must have lifted the "ill shaped stone."

Again, as to the animus of the letter that Dr. Hopkins has seen fit to publish in your paper, we do not have to go far to discover it. I ascertained during the first visit of the patient that so far from endeavoring to discourage this insane *penchant* of his cousin he was desirous of keeping it up, and I told him that such was my belief. He pretended to discourage it but in reality he did not. How he profited by it I do not know although I have my suspicions; possibly his pride was flattered in some way.

But at about the time that he was writing to you, the patient was writing to me, and the following is what he wrote. It is so diametrically opposite to the impression sought to be conveyed by the Doctor that I quote an extract:

"September 14th, 1884.

"DEAR DOCTOR:—If you remember upon my leaving New York you directed me to write you upon my return relative to my condition.

"I have ignored Dr. H.'s existence since my return and am nearly broken of any inordinate desire to see him. He is at present in Thomasville near the Florida line.

"It was a severe trial to leave him but I came through, etc., etc.

"Respectfully,

[Name and post-office of patient suppressed by the EDS.]

My censure of Dr. Hopkins for not doing all in his power to discountenance his cousin's fears and delusions and the above letter are sufficient to disclose his motive for his disgraceful conduct.

But this is not quite all.

That Dr. Hopkins did not think I had acted in any way improperly is sufficiently shown by his bringing the patient to me four times after the first visit. If I had shown so improper a sense of what is right on his first visit, he certainly, if he is a gentleman, would not have entered my doors again. So it was, all an afterthought. The letter started him.

Besides, on his last visit he told me that he was himself suffering from pain in the head, vertigo, confusion of ideas, mental irritability, etc., all going to show the existence of cerebral disorder. I examined him in the same way as I had his cousin, except as to the blood, and I found that he was suffering from serious brain trouble and I prescribed a course of treatment for him. Now this was at the fifth visit. If I had maltreated his cousin is it conceivable that he would have placed himself in my hands?

And in conclusion I have to say that I would not have noticed his communication had it not appeared in a respectable Medical Journal whose Editors have received my respect.

WILLIAM A. HAMMOND, M. D.

